# 548. Combined Results of the Phase 2a/2b/3 Randomized Controlled Trials of Ensitrelvir for the Treatment of COVID-19 Infection

**DOI:** 10.1093/ofid/ofad500.617

**Published:** 2023-11-27

**Authors:** Yuko Tsuge, Hiroshi Yotsuyanagi, Norio Ohmagari, Yohei Doi, Masaya Yamato, Takumi Imamura, Takuhiro Sonoyama, Takao Sanaki, Hiroshi Mukae

**Affiliations:** Shionogi, Osaka, Osaka, Japan; The University of Tokyo, Tokyo, Tokyo, Japan; National Centre for Global Health and Medicine, Shinjuku, Tokyo, Japan; Fujita Health University School of Medicine, Toyoake, Aichi, Japan; Rinku General Medical Center, Izumisano, Osaka, Japan; Shionogi, Osaka, Osaka, Japan; Shionogi & Co., Ltd., Osaka, Osaka, Japan; SHIONOGI & CO., LTD., Toyonaka-shi, Osaka, Japan; Nagasaki University, Nagasaki, Nagasaki, Japan

## Abstract

**Background:**

Ensitrelvir is a selective SARS-CoV-2 3CL protease inhibitor developed as an oral therapy for the treatment of COVID-19 infection. We report the combined results for ensitrelvir 125 mg and 250 mg treatment from the Phase 2a, 2b, 3 parts of the Phase 2/3 study conducted in patients with COVID-19 with or without SARS-CoV-2 vaccination and risk factors for severe disease.

**Figure:**
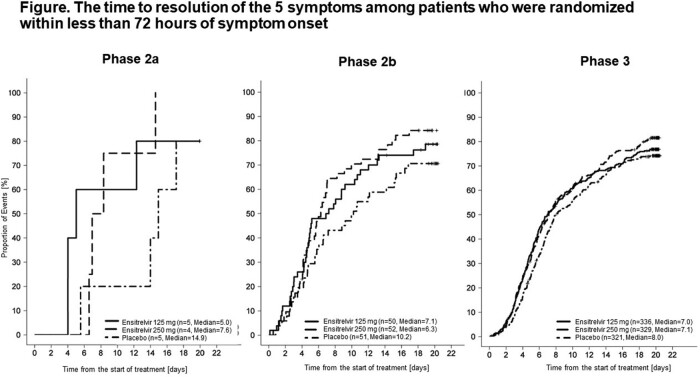

**Methods:**

The multicenter, randomized, double-blind, placebo-controlled studies were conducted between September 2021 and August 2022 in Japan, South Korea and Vietnam. SARS-CoV-2-positive patients, aged 12–70 years old, were treated with ensitrelvir 125 mg PO (after a loading dose of 375 mg PO on Day 1 only), 250 mg PO (after a loading dose of 750 mg on Day 1only) or placebo, once daily for 5 days, and followed up for 28 days from the start of treatment. Time to resolution of 5 symptoms of COVID-19 (runny or stuffy nose, sore throat, cough, feeling hot or fever, low energy or tiredness) primarily among patients who were randomized within less than 72 hours of symptom onset and safety were assessed.

**Results:**

In this post-hoc analysis, among patients who were randomized within less than 72 hours of symptom onset, 56.0% of patients were male in the ensitrelvir 125 mg group (N=407), 54.3% in the 250 mg group (N=398) and 52.5% in the placebo group (N=402). The mean (standard deviation) age was 35.8 (12.7) years in the 125 mg group, 35.1 (12.3) in the 250 mg group and 34.9 (12.3) years in the placebo group. The time to resolution of the 5 symptoms was significantly shorter in the 125 mg group (P=0.0120) compared with the placebo group (median: 167.9 hours in the 125 mg group [N=391], 167.8 hours in the 250 mg group [N=385], and 204.4 hours in the placebo group [N=377]). For patients who were randomized from onset within 120 hours, the median time to resolution was 183.2 hours in the 125 mg group (N=702), 168.9 in the 250 mg group (N=697) and 206.3 hours in the placebo group (N=692) (P=0.1235). One (0.1%) patient in the 125 mg group and 3 (0.4%) patients in the placebo group had serious adverse events; none resulted in death. No serious adverse events were observed in the 250 mg group.

**Conclusion:**

Treatment of patients with COVID-19 with ensitrelvir significantly shortened the time to symptom recovery. Ensitrelvir was well tolerated.

**Disclosures:**

**Yuko Tsuge, MSc**, SHIONOGI & CO., LTD.: Stocks/Bonds **Hiroshi Yotsuyanagi, MD PhD**, Shionogi: Advisor/Consultant|Shionogi: Advisor/Consultant **Yohei Doi, MD, PhD**, bioMerieux: Advisor/Consultant|FujiFilm: Advisor/Consultant|Gilead: Advisor/Consultant|Gilead: Honoraria|GSK: Advisor/Consultant|Meiji Seika Pharma: Advisor/Consultant|Moderna: Advisor/Consultant|Moderna: Honoraria|MSD: Advisor/Consultant|MSD: Honoraria|Shionogi: Advisor/Consultant|Shionogi: Grant/Research Support|Shionogi: Honoraria **Masaya Yamato, MD, PhD**, Shionogi: Advisor/Consultant **Takuhiro Sonoyama, MD**, SHIONOGI & CO., LTD.: I am an employee of SHIONOGI & CO., LTD.|SHIONOGI & CO., LTD.: Stocks/Bonds

